# Identifying and Prioritizing Family Education Needs at Pediatric Oncology Centers in Central America and Mexico

**DOI:** 10.1200/JGO.19.00272

**Published:** 2019-12-13

**Authors:** Erin McCann, Soad Fuentes-Alabí, Federico Antillón, Lourdes Vega-Vega, Maria Sabina Sanchez, Irini Albanti

**Affiliations:** ^1^ Harvard T.H. Chan School of Public Health, Boston, MA; ^2^ Benjamin Bloom National Children’s Hospital, San Salvador, El Salvador; ^3^ Unidad Nacional de Oncología Pediátrica and School of Medicine Francisco Marroquin University, Guatemala City, Guatemala; ^4^ Hospital Infantil Teletón de Oncología, Querétaro, Mexico; ^5^ Hospital del Niño Dr. José Renán Esquivel, Panama City, Panama; ^6^ Dana-Farber/Boston Children’s Cancer and Blood Disorders Center, Boston, MA

## Abstract

The educational needs of parents at the time of their child’s cancer diagnosis are often unclear, and research on this topic in low- and middle-income countries is limited. This study evaluated the educational needs of families at major pediatric oncology centers in Central America and Mexico.

**METHODS:**

A qualitative study involving 72 in-person interviews and 4 focus groups was conducted using a semistructured interview guide. Key informants included family members, physicians, nurses, psychosocial providers, foundation leadership, volunteers, and communication professionals. The study sites included pediatric oncology centers in El Salvador, Guatemala, Mexico, and Panama. NVivo was used for thematic analysis.

**RESULTS:**

Across all sites, parents had common questions and educational needs. Questions from families focused on their child’s likelihood of dying from cancer and feelings of guilt that were based on their perception that they caused the disease. The origin of cancer, nutrition, and psychosocial support were the most important educational themes. However, the prioritization of different educational themes varied on the basis of cultural or social influences unique to each site. Some of these differences included a need for education surrounding amputations, sibling support, and alternative or traditional healers.

**CONCLUSION:**

This study demonstrates that although many educational needs were consistent across hospitals, some of the educational priorities differed by site despite geographic proximity and shared language. Developing an educational program in resource-limited settings can be challenging, but it is an important contributor to improving childhood cancer outcomes that should be tailored to the specific needs of a site. This study can be used as a guide for other programs with limited resources wanting to develop relevant educational materials for families.

## INTRODUCTION

Eighty percent of children diagnosed with cancer each year live in low- and middle-income countries (LMICs),^1^ and the burden of disease is thought to be larger than previously estimated, with 43% of childhood cancers undiagnosed globally.^2^ LMICs also bear a disproportionate share of the mortality burden, with 90% of deaths occurring in these settings.^1,3^ Although the explanation for this disparity is multifactorial, a major issue is abandonment of treatment, which affects 50% to 60% of children in LMICs.^2^ Decreased adherence to treatment has been shown to be connected to parents’ socioeconomic status, educational background, and overall fear of treatment.^1,4-8^ Furthermore, research studies have shown that low socioeconomic status may act as a proxy for poor understanding of the seriousness of a child’s diagnosis and the importance of treatment.^9^

**CONTEXT**

**Key Objective**
The key objective of this study was to explore the educational needs of families at major pediatric oncology centers in Central America and Mexico, which have not been evaluated before.
**Knowledge Generated**
Findings revealed that across all sites, parents had common questions and educational needs. Questions from families focused on their child’s likelihood of dying from cancer and feelings of guilt that were based on their perception that they caused the disease. The origin of cancer, nutrition, and psychosocial support were the most important educational themes. However, the prioritization of different educational themes varied on the basis of cultural or social influences unique to each site. Some of these differences included a need for education surrounding amputations, sibling support, and alternative or traditional healers.
**Relevance**
Developing an educational program in resource-limited settings can be challenging, but it is an important contributor to improving childhood cancer outcomes that should be tailored to the specific needs of a site.

The educational needs of parents of children with cancer at the time of their child’s cancer diagnosis are often overwhelming and complex, and research in LMICs is limited.^10^ The Pediatric Oncology International Network for Training and Education (POINTE), a subgroup of the International Society of Pediatric Oncology and the Children’s Oncology Group (COG), have started to address this knowledge gap through research and collaboration among professionals caring for families and providing education after a diagnosis of cancer. POINTE has developed an online database to help connect health care providers globally and to share professional resources relevant for low-resource settings.^11^ The COG also shares resources with providers and promotes a family-centered educational program that occurs throughout the continuum of care and takes place in a supportive environment.^12^ The COG recently published an evidence-based educational checklist that outlines primary, secondary, and tertiary topics to cover with families.^13^ This is helpful for pediatric oncology programs, but research conducted in LMICs has highlighted certain additional factors to consider when developing educational programs, including the socioeconomic level of the parents, cultural or language barriers, and the availability of qualified personnel or resources to create informational materials.^14^

Most of the education guidelines that exist, such as those published by the COG, are geared toward high-resource settings.^13^ It has not been confirmed whether these educational guidelines and materials are relevant for pediatric oncology centers in LMICs. Although there are certainly shared needs across settings, other topics or barriers may be of higher priority for families living in an LMIC. Little research exists that assesses the specific educational needs of families caring for a child with cancer in LMICs.

With the understanding that parental education and engagement could improve treatment outcomes in LMICs,^15^ and building off the COG research that supports family-centered education, we conducted our study at 4 major pediatric oncology centers in Central America and Mexico. The goal of this study was to identify specific educational themes and priorities that could be used to develop a family-oriented educational tool in low-resource settings. Our objective was to assess the educational needs of families by understanding the most common questions parents had after their child’s cancer diagnosis and the educational topics about which it was most important for families to learn.

## METHODS

This was a qualitative study using semistructured, in-depth interviews and focus groups conducted with families of children with cancer and with health care providers between 2015 and 2018. Four different pediatric oncology centers in El Salvador, Guatemala, Mexico, and Panama were chosen for this study because they represented different locations in the region with various models of care, case burden, financial structure, and associated foundation support. Although the hospital in Panama City is situated in a high-income country, it was included in the analysis because it shares similar infrastructure and education programs with the other sites located in LMICs and also sees many patients with limited education and financial resources. A description of each study site can be found in Table 1.

**TABLE 1:**
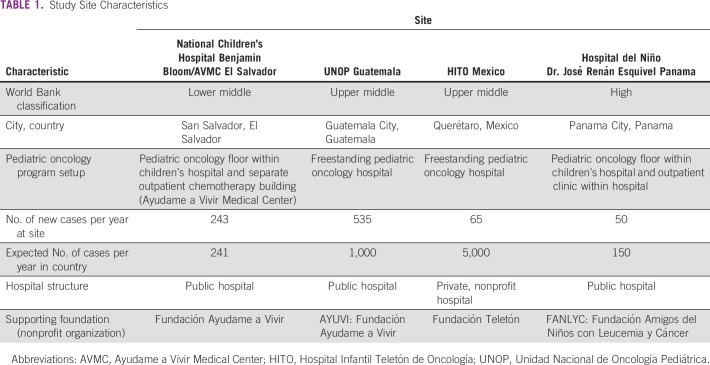
Study Site Characteristics

Any English- or Spanish-speaking adult family member of a patient diagnosed with cancer and currently receiving treatment at the study site was eligible to participate in the study. Key informants participated in individual interviews or a focus group, and they were divided into 3 separate groups, as listed in Table 2: group 1 included family members whose child was currently being treated at one of the study sites, group 2 included health care providers who cared for children with cancer at the study site, and group 3 included communication professionals. Participants were recruited for semistructured interviews through purposive and snowball sampling guided by recommendations from hospital leadership.

**TABLE 2:**
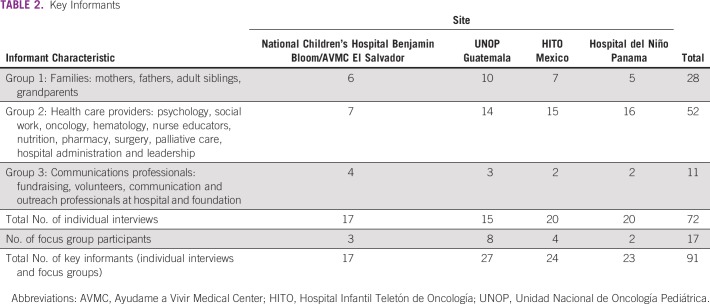
Key Informants

Interview guides were developed on the basis of a literature review of previously conducted assessments including parental education needs and parent experiences after learning their child’s diagnosis.^14-16^ Interviews were audio recorded using a mobile application, and notes were taken during the interview. Interviews were translated and transcribed into a master document, and specific quotes and common messages were categorized into broad themes. Data were categorized using a combination of Microsoft Excel and NVivo to group together repeated themes and key quotes. The most important educational themes were determined by the frequency with which the topic was mentioned throughout the interviews.

The research was approved by the institutional review board (IRB) at the Harvard T. H. Chan School of Public Health and was also approved internally at each hospital via the local IRB or hospital administration. Verbal consent was obtained before every interview. All study materials were created in English and translated into Spanish by the research team. Translated study materials were also reviewed by the IRB at Harvard Chan and by staff at each site.

## RESULTS

The most common questions identified included topics surrounding mortality, personal guilt, daily life, and financial burden. The most important educational themes were the origin of cancer, nutrition, and emotional support (Tables 3 and 4). Although all sites shared a common language and relative geographic proximity, significant differences in questions and educational preferences arose.

**TABLE 3:**
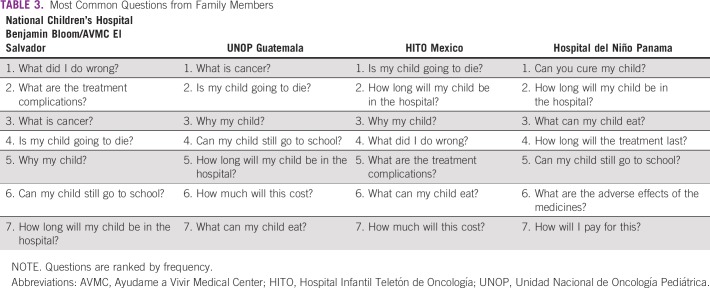
Most Common Questions from Family Members

**TABLE 4:**
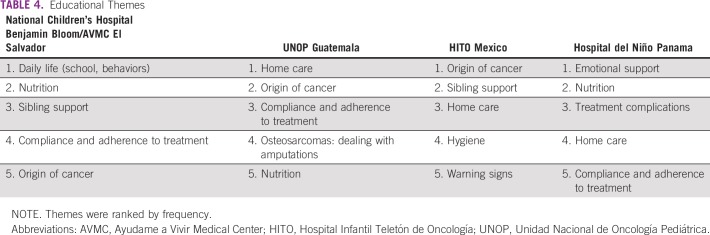
Educational Themes

### Most Common Questions/Concerns From Families

#### Mortality.

Across all four oncology centers, one of the top concerns articulated by families was the fear that their child would die after receiving the diagnosis of cancer. As one father in Guatemala described the concept of cancer in his rural town, “Cancer is considered something people die of in communities.” This concern was voiced by families and the health team in approximately 50% of all interviews, but with slight nuances. At Unidad Nacional de Oncología Pediátrica (UNOP) Guatemala and Benjamin Bloom El Salvador, the phrase, “Is my child going to die?” was repeated several times. At Hospital Infantil Teletón de Oncología (HITO) Mexico, health care providers described their community’s perception of the disease with the exact words, “Cancer equals death” in approximately one third of the interviews. At Hospital del Niño Panama, parents voiced this same question but from a different perspective, asking, “Will my child be cured?” As one pediatric oncologist from Panama described this specific phrasing of the question, “They worry a lot about whether their child will be cured. That is the first question that they practically all ask, and the way they ask the question really grabs your attention, because it is always in a positive way. They never mention the phrase, ‘Is he going to die?’ but they ask me the opposite—'Will he be cured?’”

#### Feelings of guilt.

Another common theme that was apparent was that parents, and in particular, mothers, often felt extreme guilt and responsibility for their child’s diagnosis. Parents often asked why this happened to their child and what they had done wrong while raising their child to cause the cancer. As one mother from Mexico described her feelings of guilt, “Did I do something wrong? I had never left my children without food, I always tried to keep our home clean.” A mother in El Salvador described this phenomenon by stating, “Many people think we did something wrong to cause this in our children. People don’t understand, they don’t know.”

#### Daily life.

Families also had many questions regarding the disruption to their normal daily routines. Could their child go to school, could they play with their siblings, would they have to get rid of their household pets?

#### Financial burden.

Throughout the interviews, the health care team emphasized the financial burden that is placed on families who have children undergoing treatment for cancer. After hearing the diagnosis, families often had questions about how they would be able to pay for the treatment and hospitalizations.

### Educational Themes

Key informants expressed interest in wanting to focus educational efforts on the following 3 topics.

#### Origin of cancer.

The health care teams often felt that explaining basic details about the origin of cancer as a disease was the most important topic to cover first. This concept was emphasized most at UNOP Guatemala and Benjamin Bloom El Salvador. As a psychologist in Guatemala described, “We try to explain in simple words, simple pictures, what cancer is, how it appears and how it is diagnosed and treated, so that they have this first idea of what might be happening with their child. One of the biggest needs of people is to understand what cancer is; like debunk all the myths that are around cancer.” A palliative care physician in Guatemala discussed how some of her patients believe cancer has “magic origins,” and that a family may believe their child has cancer because of the “malhecho” (bad deed) or “mal ojo” (bad eye) caused by another family. She continued, “It would be nice to have a video to talk about cancer, the origin of it, the importance of treatment.”

#### Nutrition.

Families often had questions about what to feed their child after starting treatment, and the health care team emphasized the importance of healthy nutrition. At the supporting foundation Fundación Amigos del Niños con Leucemia y Cáncer in Panama, one of their large educational campaigns was titled “4U.” This was a campaign made up of 4 pillars of education, with 2 of the pillars including “what I eat” and “how I move.” Nutrition was one of the topics that could be most distressing for families; as one grandmother described it, “The problem with my little girl is that she won’t eat. And she’s very choosy with what she eats.” Health professionals recognized the cultural importance of food for families, so nutritionists and other providers tried to prepare families for different phases of treatment when children might have different preferences for foods or might not be able to eat at all.

#### Psychosocial and emotional support.

Finally, one of the most important educational themes echoed by parents and the health care team was the emphasis on psychosocial education, from family support (including parents and siblings) to stress relief and relaxation. Parents repeated over and over again how after hearing their child’s diagnosis for the first time their mind was “blocked” or they were “in shock.” One mother described it as if her “mind was in the clouds.” The reliance on family support during this difficult time was emphasized heavily as one father described, “I think that the family is what matters most.” One nurse further echoed this sentiment saying, “There is a tremendous impact, an emotional impact. In this moment, the family needs a lot of support.” One of the leaders at Fundación Amigos del Niños con Leucemia y Cáncer, the supporting pediatric cancer foundation in Panama, recognized the need for emotional support of their families and said, “The theme of stress and emotions are the 2 themes that we are always looking for professionals to come and work with our families.” The other 2 pillars of the “4U” campaign mentioned previously included stress management and spirituality as primary education priorities.

### Differences Across Sites

The questions and educational themes mentioned by health professionals and families often overlapped, but interesting differences were noted across sites, dictated by local cultural customs or beliefs in 3 main areas, as follows:

#### Amputations.

A unique theme mentioned in multiple interviews at UNOP Guatemala and several times at HITO Mexico was the concept of amputations for children with advanced osteosarcoma. Although this type of cancer accounts for < 5% of patients at UNOP Guatemala, it creates a significant issue when families refuse amputations because of cultural beliefs of alternative remedies or fear of social isolation after the procedure. Psychologists and physicians at UNOP Guatemala were insistent that an educational tool needed to be developed to address this issue that would be relevant to families both culturally and linguistically.

#### Sibling support.

At Benjamin Bloom El Salvador and HITO Mexico, health professionals and families asked for more educational resources geared toward the siblings of patients, because it was difficult for these children to understand why their brother or sister was suddenly receiving so much attention from their parents. With the unique setup at HITO Mexico, where a parent or guardian remained with the patient at a hospital-sponsored apartment on the same hospital campus throughout the entire duration of treatment, sibling support was especially emphasized as an educational need because siblings often felt neglected at home when parents went to live with their other child at the hospital.

#### Alternative therapies.

The use of alternative remedies or practices before or in conjunction with medical treatment was described frequently at HITO Mexico as well as at Hospital del Niño Panama and was considered to be a barrier to medical treatment. As one oncologist described, “I think it’s also easier to believe in something, it gives you more hope…Here, it’s a lot about faith… And of course, as doctors, we try to put a link between alternative treatments, so that you can incorporate the faith. Because if the patient and the family lose hope, it’s another thing you have to fight with. They can’t lose this ‘drive,’ I’m not sure how else to call it.” Another physician at UNOP Guatemala described her worry that, “People believe sometimes that faith is sufficient enough to cure them, and they abandon treatment.*”*

## DISCUSSION

This study shows that although educational needs were often similar across multiple sites, several differences arose in common questions and prioritization of educational information. Families asked about their child’s prognosis and who was to blame for the disease. They wanted to know how they could keep living their regular lives and how they would pay for treatment. Providers wanted to teach families about the origin of cancer and the importance of clean nutrition and to emphasize the importance of mental health and emotional support.

This project acts as a strong reminder that educational needs should be investigated thoroughly on a local scale and in a local context, because each site had differences in the prioritization of certain topics despite geographic proximity and a shared language. For example, as mentioned previously, a top educational need at UNOP Guatemala was to teach parents about the need for amputation if their child has advanced osteosarcoma, even though this was not as common a diagnosis in their patient population. Health professionals described their strategies to address this need, such as showing YouTube videos of American Paralympians, but it was difficult for Guatemalan families to relate to these videos. This is an example of an educational need that arose as a result of a unique cultural stigma against amputations, and it is important to develop a tool that specifically targets this population with this educational need in mind.

**FIG 1:**
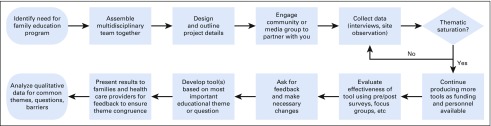
Process map.

In considering the implementation and practice implications of the results of this study, the research team created a process map (Fig 1) describing the development of educational tools for a specific site. This guide outlines each step, from identifying an educational need to conducting a needs assessment and testing the relevance and validity of an educational tool. Other hospitals may use this guide within the field of pediatric oncology or apply it more broadly to any pediatric condition.

This study has several strengths and limitations that should be addressed. Three of the 4 sites are main referral centers for the entire country and therefore see a broad and diverse patient population. These clinicians have also built strong networking relationships among themselves to share knowledge and are actively interested in supporting the work of each other to improve family education. However, there are several limitations that could be evaluated further in subsequent research. All interviews were conducted within a relatively short time frame. The families and professionals interviewed were referred to the researchers through hospital administration or personnel, creating a convenient but limited sample. The proportion of total cancer cases seen at each site varied widely, from < 5% of all cases in the country at HITO Mexico to > 90% at Benjamin Bloom El Salvador. This could have affected the perspectives of families and therefore is not fully representative. The socioeconomics of a country may affect some of the differences in perspectives, too. Several families from Hospital del Niño Panama seemed to have had more baseline knowledge about cancer and were more realistic about its causes and consequences. Another aspect that was not documented clearly was the amount of time between the initial diagnosis and the interview with a family. This could influence a family’s educational priorities or emotional burden, and therefore affect insight into their child’s diagnosis. Finally, no patients were interviewed because of ethical and feasibility concerns, but it would be important to consider the perspective of adolescents and young adults who are often independent and active participants in the educational activities throughout their treatment.

This study highlights the various educational needs at different pediatric oncology centers in El Salvador, Guatemala, Mexico, and Panama. It is important to support research in LMICs to understand the educational needs of families whose child has recently been diagnosed with cancer. A one-size-fits-all tool is not appropriate in pediatric oncology because the educational priorities of families differ on the basis of education, culture, and socioeconomic factors. This study demonstrated differences in the questions posed by parents, the educational needs at particular sites, and the barriers that exist to educating families. Especially in LMICs where the rate of abandonment is much higher than in high-resource settings,^4^ it is important to closely involve the family in their child’s care so that they understand the barriers to adhering to treatment. Innovative and engaging education that addresses the most important concerns of families should be developed to enhance the understanding of this complex and overwhelming disease. Families are the most important stakeholder, and active engagement in their child’s care can decrease the disparities that currently exist in pediatric oncology care in LMICs.
